# Prevalence and economic burden of depression and anxiety symptoms among Singaporean adults: results from a 2022 web panel

**DOI:** 10.1186/s12888-023-04581-7

**Published:** 2023-02-14

**Authors:** Parth Chodavadia, Irene Teo, Daniel Poremski, Daniel Shuen Sheng Fung, Eric Andrew Finkelstein

**Affiliations:** 1 Duke University School of Medicine, 8 Searle Center Drive, Durham, NC 27705 USA; 2 Lien Centre for Palliative Care, Duke-NUS Medical School, 8 College Rd, Singapore, 169857 Singapore; 3 Institute of Mental Health, Singapore, Singapore; 4 Department of Developmental Psychiatry, Institute of Mental Health, Singapore, Singapore; 5 Duke-NUS Medical School, 8 College Rd, Singapore, 169857 Singapore

**Keywords:** Mental health, Depression, Anxiety, Coronavirus, Cost of illness, Presenteeism, Absenteeism, Singapore

## Abstract

**Background:**

Major depressive disorder (MDD) and generalized anxiety disorder (GAD) are leading causes of disability and premature mortality. At a global level, over 300 million people are estimated to suffer from major depressive disorders, equivalent to 4·4% of the world’s population. Pandemic era stressors have increased rates for depression and anxiety by upwards of 25%. The goal of this study is to estimate the prevalence and economic burden of depression and anxiety symptoms in Singapore after the peak of the COVID-19 pandemic.

**Methods:**

An existing web panel was queried between April 2022 and June 2022. Adult participants aged > 21 years old who screened positive for depression and anxiety symptoms based on the Patient Health Questionnaire-4 (PHQ-4) Screener were eligible for participation. Prevalence estimates were quantified by dividing the number of respondents who screened positive for these symptoms by the total number of respondents. Participants who screened positive were asked about healthcare utilization, days missed from work, and reduced productivity due to these symptoms. These values were then monetized and scaled based on prevalence and population counts to generate per capita and total annual costs.

**Results:**

Two thousand three hundred forty-eight respondents filled out the PHQ-4 depression/anxiety screener on behalf of the 5,725 adults living in their households (including respondents themselves). Prevalence estimates were calculated based on the responses recorded for these 5,725 adults. 14.1% adults had symptoms consistent with depression and 15.2% had symptoms consistent with anxiety. In total, 20.0% may experience symptoms consistent with at least one of these two conditions, yet approximately half reported never being formally diagnosed. 350 respondents screened positive for depression or anxiety symptoms and thus were eligible to fill out the healthcare utilization, presenteeism, and absenteeism survey. Direct annual healthcare costs due to depression and anxiety symptoms averaged Singapore dollar (SGD) $1,050 for these respondents. The employed subset (*n* = 304) missed an extra 17.7 days of work on average per year, which translates to SGD $4,980 per worker. These workers also reported being ~ 40% less productive at work, which equates to SGD $28,720 in economic losses annually. In total, these symptoms caused SGD $15.7 billion in increased costs. Presenteeism accounts for 81.6% of this total (SGD $12.8 billion), absenteeism for 14.2% (SGD $2.3 billion) and healthcare accounts for 4.2% (SGD $0.7 billion).

**Conclusions:**

The health and economic burden associated with depression and anxiety symptoms is large in Singapore, representing 2.9% of Singapore’s gross domestic product (GDP). Employers and governments should look to identify effective remediation strategies, including strategies to address the high rates of undiagnosed cases. Increasing psychiatric resources, general practitioner mental health competency, access to peer support, and increased efforts to reduce mental health stigma should be considered to address this growing public health crisis.

**Supplementary Information:**

The online version contains supplementary material available at 10.1186/s12888-023-04581-7.

## Introduction

Major depressive disorder (MDD) and generalized anxiety disorder (GAD) are leading causes of disability and premature mortality. At a global level, over 300 million people are estimated to suffer from major depressive disorders, equivalent to 4.4% of the world’s population [1]. A similar number of people suffer from anxiety disorders, often with co-occurring depression. Depression is ranked by the World Health Organization (WHO) as the single largest contributor to global disability (7.5% of all years lived with disability in 2015), while anxiety ranks 6^th^ (3.4%). The high disease burden further strains health systems, increases rates of absenteeism and reduces productivity while working (termed presenteeism). Lost productivity alone for depression and anxiety has been estimated to cost the global economy US$ 1 trillion per year and is forecast to reach $16 trillion by 2030 [2]. Prior studies from the US estimated the economic burden of diagnosed depression to be 1.6% of its Gross Domestic Product (GDP). A systematic review estimated the burden of diagnosed anxiety disorder to be between 0.25% and 0.78% of a country’s GDP.

As alarming as these figures are, they predate the COVID-19 pandemic, which continues to have a major detrimental effect on mental health worldwide [3]. This is due both to the direct and indirect health effects of the pandemic on mental health [3]. Direct effects include SARS-CoV-2-mediated acute and long-lasting neuropsychiatric sequelae in affected individuals such as fatigue, cognitive impairments, sleep disturbance, and other health conditions that may last for unknown durations (i.e., long COVID). All of these factors may exacerbate or contribute to anxiety and depressive symptoms [4]. Indirect effects may result from social isolation and stress to the household caused by the economic downturn. Meta-analyses on the impact of COVID-19 consistently show increases in risk factors for and rates of mental health problems [5–7]. WHO estimates that COVID-19 has directly or indirectly contributed to an additional 53.2 million cases of depression and 76.2 million cases of anxiety, an increase of 28% and 26% in prevalence, respectively, since the start of the pandemic [8].

Policy makers require timely information on prevalence and economic burden of disease to prioritize prevention and treatment efforts. In the United States, early estimates of the social and economic impact of the pandemic identified target areas to alleviate future physical and mental health declines [9]. The gold-standard approach for measuring prevalence and burden of mental health conditions is through household surveys. However, this is time consuming, costly, prone to social desirability bias, and increasingly difficult to implement as home data collectors are often unwelcome, especially during pandemics when visitors in general are discouraged [10]. Reliance on healthcare administrative or billing data is problematic because many individuals with mental health conditions remain untreated and undiagnosed, and this may be exacerbated during periods of general quarantine [11]. This is especially true in countries where stigma may disproportionately keep individuals from seeking mental health treatment [12].

The goal of this study is to estimate the prevalence and economic burden of diagnosed and undiagnosed depression and anxiety in Singapore via the use of an existing web panel. This study is timely as no such estimates currently exist in the city-state. Although not without limitations, this expedient and low-cost approach generates timely information for policymakers. Due to high rates of undiagnosed mental health conditions, we focus on the economic burden of self-reported depression and anxiety symptoms, which can be obtained via use of existing screening instruments. To estimate the total economic burden of these symptoms, we examine three key measures of burden: healthcare expenditures based on monetizing the value of self-reported healthcare utilization data; absenteeism based on the market value of self-reported days missed from work due to depression or anxiety symptoms; and presenteeism, which is the monetized value of self-reported reductions in productivity while working resulting from these symptoms. We focus on Singapore, where data on post-pandemic prevalence and economic burden of mental health conditions is lacking, but the approach is generalizable as existing web panels are now available in countries worldwide.

## Methods

### Data collection

To quantify the economic burden of depression and anxiety symptoms, a cross-sectional online survey was administered in English, the official language in Singapore, to residents who are members of a national web panel curated by Kantar Profiles Division. Online surveys were chosen over interview-administered methods because of its potential to reduce social desirability bias [13]. Panel participants are recruited country wide to take surveys on a regular basis. They can remain on the panel for as long as they wish. Participants receive incentives in the form of redeemable points for select rewards. Most households remain on the panel for 2–3 years. In Singapore, the panel exceeds 500,000 individuals and is broadly representative of the socioeconomic, gender, and ethnicity distributions in Singapore.

### Participants, screener, and sample size

Participants were recruited between April 20, 2022 and June 1, 2022 through email invitations to panel members via convenience sampling. Participants were then provided with a link to the Patient Health Questionnaire-4 (PHQ-4) screener. The PHQ-4 is the combination of the Patient Health Questionnaire-2 (PHQ-2) and Generalized Anxiety Disorder 2-item (GAD-2), which are validated two-item, two-week recall period, ultra-brief screeners shown to have high sensitivity (83% and 88% respectively) and specificity (90% and 82% respectively) for assessing likely depression or anxiety [14]. Increasing PHQ-4 scores have been shown to be strongly associated with multiple domains of functional impairment, disability days, and healthcare use. While the PHQ-4 is not a diagnostic tool for depression or anxiety, the brevity of the survey and high sensitivity and specificity allow for rapid identification of both diagnosed and undiagnosed individuals who are likely to have these conditions.

Participants were eligible to fill out the screener if they were a Singaporean citizen or permanent resident over age 21. In total, 2,348 respondents filled out the PHQ-4 depression/anxiety screener on behalf of the 5,725 adults living in their households (including respondents themselves). Survey respondents were asked to fill out the full survey if they or another adult household member experienced at least mild symptoms of major depressive disorder and/or generalized anxiety disorder as indicated by the PHQ-4 (score greater than or equal to 3 for any sub-component) [14].

Any respondent who had a history of psychiatric illness with the exception of depression or anxiety was excluded from analysis due to concerns that these other conditions would inflate the burden estimates. Based on the PHQ-4 responses, 350 respondents filled out the full survey for themselves and 79 fill out the survey on behalf of another household member as a proxy. This manuscript focuses exclusively on the 350 adults who self-reported depression or anxiety symptoms due to concerns of bias with the proxy responses as evidenced by much higher cost estimates (see Table S2). Institutional Review Board (IRB) approval (IRB # 2021–836) was obtained from the National University of Singapore IRB Board in March 2022.

### Measures and estimation

The full survey is available in Additional file 1: Supplementary Appendix A and included the following domains: mental healthcare utilization, productivity losses from absenteeism and presenteeism, other mental health conditions, additional socio-demographic questions. As noted above, a household member was assumed to have depression symptoms if they scored 3 or higher on the depression sub-scale (sum of items 3 and 4). Similarly, a household member was assumed to have anxiety if they scored 3 or higher on the anxiety sub-scale (sum of items 3 and 4) [14]. Prevalence rates were calculated by dividing the number of adults who score 3 or greater on the depression or anxiety sub-scale of the PHQ-4 by the total number of reported adults across all households in the study. We present separate prevalence estimates for symptoms of depression (with or without anxiety) and anxiety (with or without depression) and present the percentage of each group who report being undiagnosed, defined as never been told by a healthcare professional that they have the condition.

To quantify costs of healthcare utilization attributable to depression and anxiety symptoms, questionnaires included content about the frequency of physician and outpatient visits (including tele-visits) and the use of medications and alternative therapies (e.g., acupuncture, reflexology). These questions were based on the validated Medical Expenditures Panel Survey [15]. For these questions, the recall period was three months. Other questions focused on diagnostic tests, emergency department visits, and number and duration of hospitalizations. For these we used a recall period of twelve months, as these episodes are less frequent and easier to remember with longer recall periods. To monetize healthcare utilization, unit costs were applied to each type of service based on unsubsidized costs collected through publicly available sources. Full breakdown of unit costs and assumptions are available in Table S3. Per capita healthcare cost estimates were taken by averaging across respondents. Total cost estimates were generated by multiplying Singapore adult population counts from the Department of Statistics times our estimated prevalence rates times the per capita cost estimates. Given the high variance in the per capita estimates and results showing that symptoms often co-occur, we present all burden estimates for depression and anxiety symptoms combined as opposed to separate estimates for each condition.

Lost productivity was quantified using a modified version of the Workplace Productivity and Activity Impairment Questionnaire: Specific Health Problem V2 fielded to the subset of respondents (*n* = 304) who reported full- or part time employment [16]. Absenteeism was captured by asking these respondents to indicate the number of hours missed from work *due to problems associated with symptoms of depression and/or anxiety* in the past week. This figure was then multiplied by 48 (number of weeks in a work-year) to generate annual hours missed in a year and monetized by multiplying by an average hourly wage estimate for each respondent. For full time employees (160 + monthly hours), hourly wages were calculated by dividing reported monthly income by the sum of reported number of monthly hours worked and monthly hours missed from work. Monthly income was assumed to be the midpoint of the reported income category or Singapore dollars (SGD) $15,500 for those who report earnings in SGD $15,000^1^ or more category. There was no missing data among primary respondents for monthly income.

Presenteeism was captured based on a question asking employed respondents the degree to which depression and/or anxiety symptoms affected productivity while working on a scale of 0–10 with 0 being “no symptoms and/or symptoms had no effect on my work” and 10 being “symptoms completely prevented me from working”. Monthly presenteeism hours were calculated as the product of a participants’ presenteeism scale response and their reported monthly number of hours worked. This estimate was then annualized and monetized using an analogous approach as for absenteeism to generate per capita costs. As only those in the labor force can generate absenteeism and presenteeism costs, we estimated total costs as the product of number of adults employed in the labor force in Singapore (again from the Department of Statistics) times our estimates of the prevalence of depression and/or anxiety times the estimated unit cost estimates of absenteeism and presenteeism. All costs are reported in 2022 Singapore dollars [1].

Before conducting any analyses, data was cleaned and non-logical answers were re-coded as missing (e.g., reporting missing more hours from work than there are hours in a week). Details on data cleaning are available in Additional file 1: Supplementary Appendix B. All analyses were conducted in Stata/SE 17.0 (College Station, Texas, United States).

## Results

### Prevalence of depression and anxiety

In total, 2,348 respondents filled out the PHQ-4 depression/anxiety screener on behalf of the 5,725 adults living in their households (including respondents themselves). Out of the 5,725 adults, 14.1% were reported to have symptoms consistent with depression and 15.2% have symptoms consistent with anxiety. Based on the PHQ-4 responses, 66.0% of those with depression also had symptoms consistent with anxiety. Similarly, 61.0% of those with anxiety also had symptoms consistent with depression. In total, 20.0% were reported to have at least one of these two conditions yet roughly half of these (53.6% for anxiety and 49.1% for depression) reported never being formally diagnosed with depression or anxiety.

### Respondent characteristics

Characteristics of survey respondents with depression or anxiety symptoms (*N* = 350) and those of the general population of adults in Singapore can be found in Table 1. Those reporting mental health conditions are younger, less likely to be married, more likely to have achieved university level education, more likely to be part-time employed, and more likely to earn higher monthly incomes compared to general residents in Singapore. This is due partly because web panels skew toward younger populations but likely also because there is a higher incidence of mental health conditions among working-age adults [17].

**Table 1:** Demographic and Clinical Characteristics of Primary Respondents by PHQ-4 Status (*N* = 350)^a^

Category	Depression (*N* = 248)	Anxiety (*N* = 269)	Singapore Residents
***Demographic Characteristics***			
**Mean Age**	36.9 (SD: 12.8)	37.4 (SD: 11.3)	49.2 (SD: 16.8)
**Female (%)**	126 (50.8%)	149 (53.9%)	1,628,591 (51.5%)
**Chinese (%)**	201 (81.1%)	217 (80.7%)	2,400,913 (76.0%)
**Married (%)**	114 (46.0%)	140 (52.0%)	2,035,800 (64.2%)
**Education Level (%)**^**b**^			
Primary to Junior College	112 (45.1%)	120 (44.6%)	1,904,000 (63.8%)
University and Above	135 (54.4%)	149 (55.4%)	1,074,300 (36.1%)
**Employment Status (%)**			
Full-Time	115 (46.3%)	127 (47.2%)	1,862,500 (59.0%)
Part-Time	104 (41.9%)	102 (37.9%)	220,975 (7.0%)
Not Employed	29 (11.7%)	40 (14.9%)	1,073,304 (34.0%)
**Monthly Income (%)**			
No Income	29 (11.7%)	40 (14.9%)	1,039,756 (32.9%)
SGD 0 to SGD 1999	23 (9.3%)	24 (9.0%)	497,000 (15.7%)
SGD 2000 to SGD 4999	103 (41.5%)	103 (38.0%)	854,560 (27.1%)
SGD 5000 to SGD 9999	74 (29.8%)	82 (31.0%)	508,320 (16.1%)
SGD 10,000 +	19 (7.7%)	20 (7.0%)	259,460 (8.2%)
***Clinical Characteristics***			
**Mean EQ5D Score**	0.77 (SD: 0.25)	0.75 (SD: 0.25)	N/A
Anxiety and Depression Sub-Score	2.21 (SD: 1.00)	2.08 (SD: 0.99)	N/A
**Mean PHQ-4 Score**			
Mean Total Score	7.8 (SD: 2.7)	7.5 (SD: 2.8)	N/A
Mean Depression Sub-Score	4.2 (SD: 1.2)	3.3 (SD: 1.9)	N/A
Mean Anxiety Sub-Score	3.6 (SD: 1.8)	4.2 (SD: 1.2)	N/A

^a^Columns for each characteristic may not add up to 100% due to rounding errors. ^b^This includes junior college, the Singapore-Cambridge General Certificate of Education Advanced Level (A-Level examination, polytechnic education, diplomas, vocational training, and Institute of Technical Education (ITE) education. The A-Level is a national examination held annually in Singapore. The examination is taken by school candidates on the completion of preuniversity education at junior colleges centralised institutions, and Integrate Programmes, and is also open to private candidates. ITE is a public vocational education institution agency in Singapore that provides pre-employment training to secondary school graduates, and continuing education and training to working adults

### Annual healthcare resource utilization

A detailed breakdown of healthcare resource utilization is available in Table 2. Over the past three months, 32% of respondents reported obtaining healthcare to treat their mental health conditions. 17% reported prescription medication use while 24% consulted a mental health provider. Contextualizing mental health provider consults, 13% of respondents consulted a general practitioner at a government run polyclinic, 10% consulted a private general practitioner, 7% consulted a psychiatrist, 7% consulted a psychologist, and 4% consulted a social worker or life coach. Over the past year, 13% had at least one visit to the ED, 9% had at least one hospital admission, 7% underwent an ECG and 6% underwent an MRI as part of their diagnostic journey. Among respondents who saw a mental healthcare provider, psychologists and life coaches were most frequent. Among respondents who were admitted to the hospital, average number of admissions was 1.3 with average length of stay of 3.3 days. Approximately half of these admissions were through the ED suggesting it was in response to an acute episode. Among respondents who used diagnostic tests, EKGs were the most frequent tests (42 uses out of 142 total diagnostic tests), presumably to rule out other health concerns.

**Table 2:** Summary characteristics of healthcare utilization among primary respondents who screened positive for depression and/or anxiety

**Recall Period**	**Healthcare Cost**	**Primary Respondents (*N* = 350)**	**Healthcare Users (*N* = 109)**^**a**^	
		**Utilized Resource, N (%)**	**Frequency of Use (N)**	**Mean Use Per Person (SD)**
**3 Months**	**Medication Use**	**58 (17%)**	**-**	**-**
	Daily Medication	29 (8%)	**-**	**-**
	As Needed Medication	31 (9%)	**-**	**-**
	Insomnia Medication	21 (6%)	**-**	**-**
	Medication Not Listed	0 (0%)	**-**	**-**
	Other Medication	1 (0%)	**-**	**-**
**3 Months**	**In-Person Physician Consultations**	**83 (24%)**	**289**	**3.6 (3.1)**
	Polyclinic	44 (13%)	78	1.8 (0.9)
	Private GP	35 (10%)	52	1.5 (0.7)
	Psychiatrist	26 (7%)	47	1.8 (1.2)
	Psychologist	24 (7%)	61	2.5 (2.5)
	Social Worker	7 (2%)	25	2.1 (1.3)
	Life Coach	7 (2%)	16	2.3 (1.9)
	Other	7 (2%)	10	1.4 (1.1)
**3 Months**	**Tele-Physician Consults**	**36 (10%)**	**114**	**3.2 (2.6)**
	Polyclinic	19 (5%)	27	1.4 (0.8)
	Private GP	12 (3%)	14	1.2 (0.6)
	Psychiatrist	11 (3%)	16	1.5 (0.8)
	Psychologist	13 (4%)	27	2.1 (1.3)
	Social Worker	6 (2%)	10	1.7 (0.9)
	Life Coach	4 (1%)	12	3.0 (2.3)
	Other	3 (1%)	8	2.7 (1.5)
**1 Year**	**Hospitalization Status**	**40 (11%)**	**-**	**-**
	ED w/o Hospital Admission (Visits)	26 (7%)	33	1.3 (0.8)
	ED w/ Hospital Admission (Nights)	17 (5%)	50	2.5 (4.6)
	Direct Hospital Admission (Nights)	15 (4%)	47	2.8 (4.0)
**1 Year**	**Diagnostic Testing Status**	**47 (13%)**	**142**	**3.0 (2.6)**
	ECG	24 (7%)	42	1.8 (1.7)
	EEG	14 (4%)	29	2.1 (2.4)
	CT	18 (5%)	25	1.4 (1.0)
	MRI	20 (6%)	23	1.2 (0.4)
	Other Tests	9 (3%)	23	2.6 (2.9)
**1 Year**	**Any Healthcare Utilization**	**109 (31%)**	**-**	**-**

^a^Healthcare users refers to adults who have used any healthcare resource in the last three months of one year depending on the recall period

### Per capita direct and indirect economic burden

Per capita annualized costs are shown in Table 3 with additional details in Table S2. Direct healthcare costs due to depression and/or anxiety averaged SGD $1,050. Those employed full- or part-time missed 17.7 days on average per year due to symptoms associated with these conditions, which translates to SGD $4,980 in economic losses annually per person. The economic burden attributable to presenteeism far exceeded that of absenteeism. Adults with depression and/or anxiety symptoms who were employed full- or part-time reported an average presenteeism score of 4.1, which indicates that these individuals were ~ 40% less productive at work due to their mental health problems compared to their value if fully productive. This translates to missing an equivalent of 104 days on average per year, valued at SGD $28,720 in economic losses annually per person.

**Table 3:** Per Capita and Total Costs of Depression and Anxiety (SGD $)

Cost Category	Per Capita Costs^c^	Total Costs^c^	% Share of Total Cost
Healthcare^a^	1050 (3030)	$ 662,923,380	4.2%
Absenteeism^b^	4980 (9910)	$ 2,225,761,200	14.2%
Presenteeism^b^	28,720 (29,880)	$ 12,836,116,800	81.6%
**Total**	33,600 (35,660)	**$ 15,724,801,380**	100%

^a^Healthcare costs per capita are assumed to be identical for employed and unemployed individuals. ^b^Absenteeism and presenteeism costs are limited to individuals that are employed full-time or part-time. cTotal costs average per capita annual costs are provided for employed adults and total economic costs include employed and unemployed individuals

### Total economic burden of mental health in Singapore

Summing the health costs and productivity losses yields a total economic burden of depression and anxiety symptoms of SGD $15.7 billion. Presenteeism accounts for 81.6% of this total (SGD $12.8 billion), absenteeism accounts for 14.2% (SGD $2.2 billion) and healthcare resource utilization accounts for 4.2% (SGD $0.7 billion) (Table 3).

## Discussion

This is the first study after the onset of the pandemic to estimate the prevalence and economic burden of depression and anxiety symptoms among Singaporean adults. Although the PHQ-4 is a screening tool and not all those who screen positive would reach diagnostic criteria for a mental health condition, the fact that 20.0% screened positive for depression and/or anxiety symptoms based on their own or a proxy response is cause for concern.

To assess whether our prevalence estimates are credible, we compared them to an in-person household survey conducted by the Singapore Institute of Mental Health (IMH) aimed to quantify *diagnosed* prevalence of these and other mental health conditions prior to COVID-19. Using the WHO-CIDI 3.0, which requires a more stringent criteria to conform these conditions, they found one year prevalence of MDD to be 2.3% and GAD at 0.8%, which are markedly lower than our estimates [18]. However, household survey data from countries in the region have yielded prevalence rates between 4.8% and 8.1% for MDD and GAD, suggesting that respondents in Singapore may be under-reporting mental health conditions [19, 20]. Moreover, because only half of our sample has been formally diagnosed, a more appropriate comparison would be our estimate of diagnosed prevalence of 10%. Furthermore, social desirability bias may influence the degree to which people are willing to disclose potentially stigmatizing symptoms in face-to-face interview settings [10]. Estimates from other countries suggest that COVID-19 is responsible for roughly a three-fold increase in mental health conditions [21–25]. Once these concerns are considered, our estimates, although still on the high side based on the IMH data, appear credible [21–25].

Results further reveal that the economic burden of these conditions totals SGD $15.7 billion annually, with 95% coming from lost productivity, largely due to presenteeism as individuals with these conditions tend to continue to work but at far below their potential. This figure represents 2.9% of the total GDP in Singapore as of 2021. No prior estimates from Singapore are available for comparison. Prior to the onset of COVID-19, a study from the US estimated the economic burden of diagnosed depression to be 1.6% of its GDP [26]. A systematic review estimated the burden of diagnosed anxiety disorder to be between 0.25% and 0.78% of a country’s GDP [27]. While our estimate for depression and anxiety symptoms is higher, the rise in prevalence post COVID-19 and our inclusion of both diagnosed and undiagnosed individuals with depression and anxiety, plus a focus on symptoms as opposed to a clinical diagnosis, suggests our estimates are plausible [26].

One reason our estimates are higher than others may be due to the high costs of productivity losses, specifically presenteeism. The US study estimated that 60% of the costs of these conditions are due to productivity losses, which is much lower than our estimate of 95% [26]. Although these differences could be explained by multiple factors, a primary factor is that the US has greater reimbursement for mental health conditions and less stigma that would discourage access, so it is not surprising that direct medical costs make up a greater share of the total in the United States. Moreover, for our sample, 65% of respondents never sought care from the formal healthcare system. Whether the truth is 60%, 95%, or somewhere in between, clearly mental health is taking a significant toll on employee productivity and should be a cause for concern.

### Implications

Both employers and governments should take note of the high prevalence and costs of depression and anxiety symptoms in Singapore and look to identify effective prevention and remediation strategies. The Singapore government has taken note. They have been increasingly proactive in addressing mental health in the city-state and have already created a multiagency task force to address the growing mental health pandemic. Capitation models of care, where primary care providers are financially encouraged to engage in preventative medicine and early detection of chronic conditions, are being introduced in various settings. This capitation will address public health, mental health and social determinants of health. Funding has been put toward increasing mental health literacy amongst the general population, with special focus paid to employers and general practitioners. For example, the General Practitioner (GP-Partnership Programme at IMH seeks to provide general practitioners with the resources needed to increase their comfort and competence when treating people with mental health concerns. Public consultations are underway to establish a tiered system of care in which individuals with symptoms of depression and anxiety are first offered self, family, and workplace based support followed by progressively higher levels of care (e.g., mental health professional, clinics and hospitals) depending on the severity of symptoms [28].

Many employers have also enhanced their employee wellness programs and are revisiting their benefits programs to consider expanded mental health benefits [29]. However, more can be done. The gap between diagnosed and undiagnosed cases suggests there remains substantial unmet need of mental health treatment among community dwelling adults in Singapore. To close this gap, the healthcare system must be resourced accordingly [30]. Increasing the local training of psychiatrists, psychologists, nurses and counselors is vital, as the unique cultural context of mental illness means that such resources are hard to import from external settings [31]. It is also vital to increase the system’s ability to treat milder cases and prevent the deterioration that may accompany prolonged stress. Improving general practitioners’ competence and that of other allied health professionals and peer supporters may increase the availability of care and may help people gain strategies for managing concerns before they intensify.

Individuals with adverse childhood experiences are a particularly high-risk group. Providing added support for this group and subsequently following families with strong heritability of mental illnesses can help break the intergenerational cycle of mental health disorders [32, 33]. Efforts to reduce stigma can increase the likelihood that those with mental health concerns seek treatment. Future studies should test the public health impact of all of these efforts.

### Limitations

This study has both strengths and limitations. The primary strength is the ability to generate estimates of prevalence and economic burden of depression and anxiety in a low cost and timely manner. This anonymous method should reduce social desirability bias as interviewer-administered tools can lead to the under-reporting of mental health conditions [10]. A meta-analysis reported almost half the rate of detection in non-anonymous vs anonymous modes of screening [34]. The primary limitation is reliance on an online panel. Even though the panel is broadly representation of the national population on key demographic indicators, we cannot guarantee that our sub-sample of those with depression and anxiety symptoms is similarly representative as this depends on eligible participants willingness to take this survey. Another limitation is that the PHQ-4 may not pick up all patients with depression or anxiety symptoms given its lower sensitivity relative to the PHQ-9. However, we chose this survey for brevity. Comparisons with the few other published studies available suggests our results are credible despite this concern. An additional limitation is that we may have overestimated the economic burden attributable to absenteeism and presenteeism because we compare those with depression or anxiety symptoms to someone who is perfectly productive. However, it is likely that people without a mental health condition have reduced productivity due to other health conditions. Quantifying the reduction in productivity due to mental health conditions taking this into account would require access to a control group of workers with other health conditions who may or may not also have mental health conditions. This should be an area of future research. Future studies should aim to validate these results using alternative approaches, such as by accessing healthcare utilization and billing data or via other survey techniques.

## Conclusion

The health and economic burden associated with depression and anxiety symptoms is large in Singapore. The significant portion of untreated illness suggests more must be done to boost Singapore’s human capital to deal with those who may seek help. Increasing psychiatric resources, general practitioner mental health competency, peer support resources and greater efforts to reduce the stigma of mental health may be effective strategies to address this public health crisis.

## Supplementary Information

**Additional file 1:** **Appendix A.** Main Respondent Survey. **Appendix B.** Proxy Respondent Survey. **Appendix C.** Data Quality Check and Cleaning. **Supplementary Table S1.** Demographics and Clinical Characteristics by Respondent Status. **Supplementary Table S2.** Breakdown of Average Annual Per Capita Costs by Respondent Status. **Supplementary Table S3.** Unsubsidized Unit Cost Estimates for Healthcare Resources in Singapore.

## Data Availability

The datasets generated and/or analysed during the current study are not publicly available due restrictions for the NUS Institutional Review Board. However, de-identified data can be made available from the corresponding author if an approved IRB is in place from the requesting organization.

## Footnotes

1 SGD $1 = $0.75 on 31 Dec 2022.
