# Supplementary material for: Prevalence and economic burden of depression and anxiety symptoms among Singaporean adults: results from a 2022 web panel
Source: BMC Psychiatry. 2023 Feb 14;23:104. doi: 10.1186/s12888-023-04581-7 (PMC9925363; doi:10.1186/s12888-023-04581-7)
Supplement: Supplementary file 1 — Additional file 1: Appendix A. Main Respondent Survey. Appendix B. Proxy Respondent Survey. Appendix C. Data Quality Check and Cleaning. Supplementary Table S1. Demographics and Clinical Characteristics by Respondent Status. Supplementary Table S2. Breakdown of Average Annual Per Capita Costs by Respondent Status. Supplementary Table S3. Unsubsidized Unit Cost Estimates for Healthcare Resources in Singapore. [file 12888_2023_4581_MOESM1_ESM.docx]

**Supplementary Materials Overview**

*Table of Contents*

Appendix A. Main Respondent Survey ……………………………………………………………………….Page 2

Appendix B. Proxy Respondent Survey ………………………………………………………………………Page 14

Appendix C. Data Quality Check and Cleaning……………………………………………………………….Page 22

Supplementary Table S1. Demographics and Clinical Characteristics by Respondent Status……..………….Page 23

Supplementary Table S2. Breakdown of Average Annual Per Capita Costs by Respondent Status……......…Page 24

Supplementary Table S3. Unsubsidized Unit Cost Estimates for Healthcare Resources in Singapore……..…Page 25

**Appendix A. Main Respondent Survey**

*SCREENER PART 1: GENERAL QUESTIONS*

1. Are you a Singaporean Citizen or Permanent Resident (PR)?

| A | Yes |
| --- | --- |
| B | No |

[TERMINATE IF NO]

1. What is your current age?

|  |
| --- |

[TERMINATE IF <21]

1. How many children live in your household between ages 4 and 21?

| 1 | Zero |
| --- | --- |
| 2 | One |
| 3 | Two |
| 4 | Three |
| 5 | Four |
| 6 | Five |
| 7 | Six |
| 8 | Seven |
| 9 | Eight |
| 10 | More than eight |

[SKIP IF CODED 1 in 3]

1. Please enter the ages of all children between ages 4 and 21 living in your household from oldest age to youngest age:

|  | Enter number between 0 and 21 |
| --- | --- |
| Child #1 |  |
| Child #2 |  |
| Child #3 |  |
| Child #4 |  |

1. How many adult family members (>21 years of age) live in your household including yourself?

*[P/N: When directing to the proxy adult questionnaire and determining how many adults may be eligible, subtract one from the answer that’s selected]*

| 1 | Zero |
| --- | --- |
| 2 | One |
| 3 | Two |
| 4 | Three |
| 5 | Four |
| 6 | Five |
| 7 | Six |
| 8 | Seven |
| 9 | Eight |
| 10 | More than eight |

1. Please enter the ages of all adult family members living in your household from oldest age to youngest age.

*[P/N: When directing to the proxy adult questionnaire and determining how many adults may be eligible, subtract one from the answer that’s selected]*

|  | Must be >21 years of age |
| --- | --- |
| Adult #1 |  |
| Adult #2 |  |
| Adult #3 |  |
| Adult #4 |  |

1. Have any of the following individuals EVER been told by a physician that you have depression and/or an anxiety disorder?

|  | Yes, Depression | Yes, Anxiety Disorder | Yes, both | No |
| --- | --- | --- | --- | --- |
| Yourself | A | B | C | D |
| [Pipe in from 2a_Child]* | A | B | C | D |
| [Pipe in from 2a_Child]* | A | B | C | D |
| [Pipe in from 2a_AdultProxy]* | A | B | C | D |
| [Pipe in from 2a_AdultProxy]* | A | B | C | D |

*SCREENER PART 2: PHQ-4 Screener*

1. Over the last two weeks, how often have the following individuals been bothered by not being able to stop or control worrying?

|  | Not At all | Several Days | More than half the days | Nearly every day |
| --- | --- | --- | --- | --- |
| Yourself | 0 | 1 | 2 | 3 |
| [Pipe in from 2a_Child]* | 0 | 1 | 2 | 3 |
| [Pipe in from 2a_Child]* | 0 | 1 | 2 | 3 |
| [Pipe in from 2a_AdultProxy]* | 0 | 1 | 2 | 3 |
| [Pipe in from 2a_AdultProxy]* | 0 | 1 | 2 | 3 |

[**to pipe in based on number selected in 2a_Child & 2a_AdultProxy]*

1. Over the last two weeks, how often have the following individuals been bothered by little interest or pleasure in doing things?

|  | Not At all | Several Days | More than half the days | Nearly every day |
| --- | --- | --- | --- | --- |
| Yourself | 0 | 1 | 2 | 3 |
| [Pipe in from 2a_Child]* | 0 | 1 | 2 | 3 |
| [Pipe in from 2a_Child]* | 0 | 1 | 2 | 3 |
| [Pipe in from 2a_AdultProxy]* | 0 | 1 | 2 | 3 |
| [Pipe in from 2a_AdultProxy]* | 0 | 1 | 2 | 3 |

[**to pipe in based on number selected in 2a_Child & 2a_AdultProxy]*

1. Over the last two weeks, how often have the following individuals been bothered by feeling down, depressed, or hopeless?

|  | Not At all | Several Days | More than half the days | Nearly every day |
| --- | --- | --- | --- | --- |
| Yourself | 0 | 1 | 2 | 3 |
| [Pipe in from 2a_Child]* | 0 | 1 | 2 | 3 |
| [Pipe in from 2a_Child]* | 0 | 1 | 2 | 3 |
| [Pipe in from 2a_AdultProxy]* | 0 | 1 | 2 | 3 |
| [Pipe in from 2a_AdultProxy]* | 0 | 1 | 2 | 3 |

[**to pipe in based on number selected in 2a_Child & 2a_AdultProxy]*

[Criteria for Inclusion in the Study]

- [If the participant scores >3 when summing Q7 and Q8] OR
- [If the participant scores >3 when summing Q9 and Q10]

Qualification Prioritization:

1. Main Respondent
2. Child (if more than 1 child, oldest child takes priority)
3. Proxy Adult (if more than 1 adult, the oldest adult takes priority)

*MAIN SURVEY – PSYCHIATRIC HISTORY*

Have you EVER been told by a physician that you have any of the following psychiatric conditions?

| Schizophrenia or Other Psychotic Disorder | Yes | No |
| --- | --- | --- |
| Alzheimer’s Disease or Dementia | Yes | No |
| Bipolar or Manic Disorder | Yes | No |
| Panic Disorder | Yes | No |
| Phobias | Yes | No |
| Post-Traumatic Stress Disorder (PTSD) | Yes | No |
| Obsessive Compulsion Disorder (OCD) | Yes | No |
| Social Anxiety Disorder | Yes | No |
| Insomnia | Yes | No |

[If participant answers >0 on Q3 and responds YES to either Condition 1 (schizophrenia or other psychiatric disorder) OR Condition 2 (Alzheimer's disease or dementia) OR Condition 3 (Bipolar or Manic Disorder) listed in the psychiatric history, please proceed to the child screener study.

If participant answers 0 on Q3, YES to either Condition 1 (schizophrenia or other psychiatric disorder) OR Condition 2 (Alzheimer’s disease or dementia) OR Condition 3 (Bipolar or Manic Disorder) listed in Q6 AND >0 on Q4, please proceed to the adult screener study.

If participant answers 0 on Q3 and Q4 and YES to either Condition 1 (schizophrenia or other psychiatric disorder) OR Condition 2 (Alzheimer’s disease or dementia) OR Condition 3 (Bipolar or Manic Disorder) listed in Q6, TERMINATE STUDY.]

*MAIN SURVEY – MEDICATION HISTORY*

In the next section, you will be asked a series of questions on medications that you have taken or are currently taking to manage your depression/anxiety. We recommend that you have close access to your medications and pill bottles to ensure more accurate responses.

1. Have you **EVER** taken any prescription medications to treat your depression/anxiety?

| A | Yes, for Depression |
| --- | --- |
| B | Yes, for Anxiety |
| C | Yes, for Both |
| D | No |

[If Q1 = D, SKIP TO Q4]

1. In the **LAST 3 MONTHS**, have you taken any prescription medications to treat your depression/anxiety?

| A | Yes, for Depression |
| --- | --- |
| B | Yes, for Anxiety |
| C | Yes, for Both |
| D | No |

[If Q2 = D, SKIP TO Q4]

1. Are you taking any of the following medications? Please check all that apply.

| A | Daily antidepressant (e.g., Prozac, Lexapro) |
| --- | --- |
| B | As needed anti-depressant or anti-anxiety drug (e.g., Xanax, Klonopin) |
| C | As needed sleep medicines to help with insomnia |
| D | Medication not listed above |
| E | Other (please specify): __________ |

*MAIN SURVEY – HEALTHCARE UTILIZATION*

1. In the **LAST 12 MONTHS**, have you consulted a physician or other healthcare professional regarding your depression/anxiety?

| A | Yes |
| --- | --- |
| B | No |

[If Q4 = B, SKIP TO Q6]

1. In the LAST 3 MONTHS, have you visited (including tele-visits) any of the following healthcare providers *because of your depression/anxiety*? If yes, please enter the number of visits. For visit types that have more than one healthcare provider listed, select all that apply.

| Visit Type | Yes | No | If yes, state the # of **in-person visits** in the LAST THREE MONTHS. Leave blank if not applicable. | If yes, state the # of **tele-visits** in the LAST THREE MONTHS. Leave blank if not applicable. | Please list whether **the last visit** was “public” or “private”? |
| --- | --- | --- | --- | --- | --- |
| Polyclinic |  |  |  |  |  |
| Private General Practitioner |  |  |  |  |  |
| Psychiatrist |  |  |  |  |  |
| Psychologist |  |  |  |  |  |
| Social Worker |  |  |  |  |  |
| Life Coach |  |  |  |  |  |
| Other |  |  |  |  |  |

[If “Yes” to “Polyclinic”, automatically note that the visit was “public”]

[If “Yes” to “Private General Practitioner”, automatically note that the visit was “private”]

1. In the LAST 12 MONTHS, have you visited an Emergency Department and/or been admitted to the hospital because of your depression/anxiety?

| Visit Type | Yes | No | Specify |
| --- | --- | --- | --- |
| Emergency department visit **without** hospital admission |  |  | How many in the last 12 months? |
| Emergency department visit **with** hospital admission |  |  | How many total nights did you spend including all admissions in the last 12 months? |
| Direct hospital admission **without** an emergency department visit |  |  | How many total nights did you spend including all admissions in the last 12 months? |

1. In the LAST 3 MONTHS, have you used any of the following approaches to manage or treat your depression/anxiety?

| Therapy | Yes | No | If yes, please estimate the amount of money that you’ve spent on this therapy **in the last three months** in Singapore Dollars. |
| --- | --- | --- | --- |
| Acupuncture |  |  |  |
| Reflexology |  |  |  |
| Chiropractor Services |  |  |  |
| Professional Meditation Services |  |  |  |
| Other | Please specify: |  |  |

[If the respondent answers the amount of money spent question with a value >1,000 SGD, please provide a prompt to the respondent stating, “Can you please confirm that you spent more than 1,000 SGD in the last three months for these therapies?”]

1. In the LAST 12 MONTHS, have you undergone any of the following medical tests due to your depression/anxiety? *Please type in your response in the text box to automatically select that option.*

| Test Type | Yes | No | Please list the total number of tests taken in the last 12 months. | |
| --- | --- | --- | --- | --- |
| Electrocardiogram (EKG) |  |  |  | |
| Electroencephalogram (EEG) |  |  |  |  |
| CT/CAT Imaging |  |  |  | |
| MRI Imaging |  |  |  | |
| Other (Please Specify): |  |  |  | |

*MAIN SURVEY – ABSENTEEISM AND PRESENTEEISM (WPAI)*

1. Are you currently employed or self-employed (i.e., working for pay)?

| A | Yes |
| --- | --- |
| B | No |

[If Q1 = A, SKIP TO Q3]

1. If you are currently not employed, is it because of your depression/anxiety?

| A | Yes |
| --- | --- |
| B | No |

The following questions ask about the effect of your mental health-related symptoms on your ability work over the LAST WEEK. Please fill in the blanks or select a number, as indicated.

1. During the LAST WEEK, how many hours did you miss from work **because of problems associated with your symptoms of depression and/or anxiety**? *Include hours missed on days you did not work at all, days you went in late, days you left early, etc. because of your symptoms. Do not include time you missed to participate in this study.*

____________Hours

1. During the LAST WEEK, how many hours did you miss from work **because of any other reasons, such as vacation, holidays, time off to participate in this study**?

____________Hours

1. During the LAST WEEK, how many days did you actually work?

____________Days

1. During the LAST WEEL, how much did your depression/anxiety symptoms **affect your productivity while you were working**? *Think about days you were limited in the amount or kind of work you could do, days when you accomplished less than you would like, or days you could not do your work as carefully as usual. If your symptoms affected your work only a little, choose a low number. Choose a high number if your symptoms affected your work a great deal.*

| No symptoms and/or symptoms had no effect on my work | 0 | 1 | 2 | 3 | 4 | 5 | 6 | 7 | 8 | 9 | 10 | Symptoms completely prevented me from working |
| --- | --- | --- | --- | --- | --- | --- | --- | --- | --- | --- | --- | --- |

1. During the LAST WEEK, how much did your depression/anxiety symptoms affect your **ability to do your regular daily activities, other than work at a job**? *By regular activities, we mean the usual activities you do such as work around the house, shopping, childcare, studying. Think about times you were limited in the amount or kind of activities you could do and times you accomplished less than you would like. If your mental health symptoms affected your activities only a little, choose a low number. Choose a high number if your mental health symptoms affected your activities a great deal.*

| No symptoms and/or symptoms had no effect on my daily activities | 0 | 1 | 2 | 3 | 4 | 5 | 6 | 7 | 8 | 9 | 10 | Symptoms completely prevented me from doing my daily activities |
| --- | --- | --- | --- | --- | --- | --- | --- | --- | --- | --- | --- | --- |

1. What is your typical personal monthly employment income from all sources (including self-employed/business owner)? a

| 1 | Less than $1000 |
| --- | --- |
| 2 | $1000 - $1999 |
| 3 | $2000 - $2999 |
| 4 | $3000 - $3999 |
| 5 | $4000 - $4999 |
| 6 | $5000 - $5999 |
| 7 | $6000 - $6999 |
| 8 | $7000 -$9999 |
| 9 | $10,000 and over |
| 99 | Prefer not to answer |

*MAIN SURVEY – QUALITY OF LIFE ASSESSMENT (EQ5D-5L)*

Under each heading, please tick the ONE box that best describes your health TODAY.

Mobility

|  | I have no problems in walking about |
| --- | --- |
|  | I have slight problems in walking about |
|  | I have moderate problems in walking about |
|  | I have severe problems in walking about |
|  | I am unable to walk out |

Self-Care

|  | I have no problems washing or dressing myself |
| --- | --- |
|  | I have slight problems washing or dressing myself |
|  | I have moderate problems washing or dressing myself |
|  | I have severe problems washing or dressing myself |
|  | I am unable to wash or dress myself |

Usual Activities (e.g., work, study, housework, family or leisure activities)

|  | I have no problems doing my usual activities |
| --- | --- |
|  | I have slight problems doing my usual activities |
|  | I have moderate problems doing my usual activities |
|  | I have severe problems doing my usual activities |
|  | I am unable to do my usual activities |

Pain/Discomfort

|  | I have no pain or discomfort |
| --- | --- |
|  | I have slight pain or discomfort |
|  | I have moderate pain or discomfort |
|  | I have severe pain or discomfort |
|  | I have extreme pain or discomfort |

Anxiety/Depression

|  | I am not anxious or depressed |
| --- | --- |
|  | I am slightly anxious or depressed |
|  | I am moderately anxious or depressed |
|  | I am severely anxious or depressed |
|  | I am extremely anxious or depressed |

-
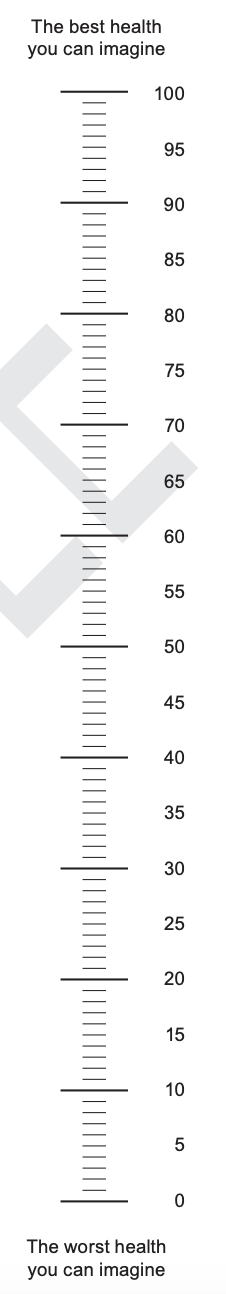
We would like to know how good or bad your health is TODAY.
- This scale is numbered from 0 to 100.
- 100 means the best health you can imagine. 0 means the worst health you can imagine.
- Mark an X on the scale to indicate how your health is TODAY.
- Now, please write the number you marked on the scale here: _______

**Appendix B. Proxy Respondent Survey**

*MAIN SURVEY – PSYCHIATRIC HISTORY*

1. You indicated that this household member has symptoms consistent with depression/anxiety. Has this person ever been diagnosed with any of the following conditions?

| Schizophrenia or Other Psychotic Disorder | Yes | No |
| --- | --- | --- |
| Alzheimer’s Disease or Dementia | Yes | No |
| Bipolar or Manic Disorder | Yes | No |
| Panic Disorder | Yes | No |
| Phobias | Yes | No |
| Post-Traumatic Stress Disorder (PTSD) | Yes | No |
| Obsessive Compulsion Disorder (OCD) | Yes | No |
| Social Anxiety Disorder | Yes | No |
| Insomnia | Yes | No |

[If participant answers YES to either Condition 1 (schizophrenia or other psychiatric disorder) OR Condition 2 (Alzheimer’s disease or dementia) OR Condition 3 (Bipolar or Manic Disorder) in Q1, TERMINATE STUDY.]

*MAIN SURVEY – MEDICATION HISTORY*

In the next section, you will be asked a series of questions on medications that this person may have taken or is currently taking to manage his or her depression/anxiety. We recommend that you have close access to this person’s medications and pill bottles to ensure the greatest level of accuracy and specificity.

1. Has this person **EVER** taken any prescription medications to treat his or her depression/anxiety?

| A | Yes, for Depression |
| --- | --- |
| B | Yes, for Anxiety |
| C | Yes, for Both |
| D | No |

[If Q1 = D, SKIP TO Q4]

1. In the **LAST 3 MONTHS**, has this person taken any prescription medications to treat his or her depression/anxiety?

| A | Yes, for Depression |
| --- | --- |
| B | Yes, for Anxiety |
| C | Yes, for Both |
| D | No |

[If Q2 = B, SKIP TO Q4]

1. Are you taking any of the following medications? Please check all that apply.

| A | Daily antidepressant (e.g., Prozac, Lexapro) |
| --- | --- |
| B | As needed anti-depressant or anti-anxiety drug (e.g., Xanax, Klonopin) |
| C | As needed sleep medicines to help with insomnia |
| D | Medication not listed above |
| E | Other (please specify): __________ |

*MAIN SURVEY – HEALTHCARE UTILIZATION*

1. In the **LAST 12 MONTHS**, has this person consulted a physician or other healthcare professional regarding his or her depression/anxiety?

| A | Yes |
| --- | --- |
| B | No |

[If Q4 = B, SKIP TO Q6]

1. In the LAST 3 MONTHS, has this person visited (including tele-visits) any of the following healthcare providers in an outpatient setting *because of his or her depression/anxiety*? If yes, please enter the number of visits. For visit types that have more than one healthcare provider listed, select all that apply.

| Visit Type | Yes | No | If yes, state the # of **in-person visits** in the LAST THREE MONTHS. Leave blank if not applicable. | If yes, state the # of **tele-visits** in the LAST THREE MONTHS. Leave blank if not applicable. | Please list whether **the last visit** was “public” or “private”? |
| --- | --- | --- | --- | --- | --- |
| Polyclinic |  |  |  |  |  |
| Private General Practitioner |  |  |  |  |  |
| Psychiatrist |  |  |  |  |  |
| Psychologist |  |  |  |  |  |
| Social Worker |  |  |  |  |  |
| Life Coach |  |  |  |  |  |
| Other |  |  |  |  |  |

[If “Yes” to “Polyclinic”, automatically note that the visit was “public”]

[If “Yes” to “Private General Practitioner”, automatically note that the visit was “private”]

1. In the LAST 12 MONTHS, has this person used any of the following services because of his or her depression/anxiety?

| Visit Type | Yes | No | Specify |
| --- | --- | --- | --- |
| Emergency department visit **without** hospital admission |  |  | How many in the last 12 months? |
| Emergency department visit **with** hospital admission |  |  | How many total nights did you spend including all admissions in the last 12 months? |
| Direct hospital admission **without** an emergency department visit |  |  | How many total nights did you spend including all admissions in the last 12 months? |

1. In the LAST 12 MONTHS, has this person used any of the following approaches to manage or treat his or her depression/anxiety?

| Therapy | Yes | No | If yes, please estimate the amount of money that you’ve spent on this therapy **in the last three months** in Singapore Dollars. |
| --- | --- | --- | --- |
| Acupuncture |  |  |  |
| Reflexology |  |  |  |
| Chiropractor Services |  |  |  |
| Professional Meditation Services |  |  |  |
| Other (Please Specify) |  |  |  |

[If the respondent answers the amount of money spent question with a value >1,000 SGD, please provide a prompt to the respondent stating, “Can you please confirm that you spent more than 1,000 SGD in the last three months for these therapies?”]

1. In the LAST 12 MONTHS, has this person undergone any of the following medical tests due to his or her depression/anxiety?  *Please type in the response in the text box to automatically select that option.*

| Test Type | Yes | No | Please list the total number of tests taken in the last 12 months. |
| --- | --- | --- | --- |
| Electrocardiogram (EKG) |  |  |  |
| Electroencephalogram (EEG) |  |  |  |
| CT/CAT Imaging |  |  |  |
| MRI Imaging |  |  |  |
| Other (Please Specify): |  |  |  |

*MAIN SURVEY – ABSENTEEISM AND PRESENTEEISM (WPAI)*

The following questions ask about the effect of this person’s mental health-related symptoms on their ability to work over the LAST 4 WEEKS, not including today. Please fill in the blanks or select a number, as indicated. Please only consider depression and/or anxiety symptoms.

1. Is this person currently employed or self-employed (i.e., working for pay)?

| A | Yes |
| --- | --- |
| B | No |

[If answer to Q1 is NO, SKIP to Q4.]

1. During the LAST 4 WEEKS, how many days did this person actually work?

____________Days

1. During the LAST 4 WEEKS, how many hours did this person miss from work **because of problems associated with their symptoms of depression and/or anxiety**? *Include hours missed on days they did not work at all, days they went in late, days they left early, etc. because of their symptoms. Do not include time you missed to participate in this study.*

____________Hours

1. During the LAST FOUR WEEKS, how much did this person’s MD/GAD symptoms **affect their productivity while they were working**? *Think about days they were limited in the amount or kind of work they could do, days when they accomplished less than they would like, or days they could not do their work as carefully as usual. If their symptoms affected their work only a little, choose a low number. Choose a high number if their symptoms affected their work a great deal.*

| No symptoms and/or symptoms had no effect on their work | 0 | 1 | 2 | 3 | 4 | 5 | 6 | 7 | 8 | 9 | 10 | Symptoms completely prevented them from working |
| --- | --- | --- | --- | --- | --- | --- | --- | --- | --- | --- | --- | --- |

1. During the LAST FOUR WEEKS, how much did their MD/GAD symptoms affect their **ability to do their regular daily activities, other than work at a job**? *By regular activities, we mean the usual activities they do such as work around the house, shopping, childcare, studying. Think about times they were limited in the amount or kind of activities they could do and times they accomplished less than they would like. If their mental health symptoms affected their activities only a little, choose a low number. Choose a high number if their mental health symptoms affected their activities a great deal.*

| No symptoms and/or symptoms had no effect on their daily activities | 0 | 1 | 2 | 3 | 4 | 5 | 6 | 7 | 8 | 9 | 10 | Symptoms completely prevented them from doing their daily activities |
| --- | --- | --- | --- | --- | --- | --- | --- | --- | --- | --- | --- | --- |

The following questions ask about the effect of this person’s symptoms on their unpaid caregiver’s (you, your spouse, other family member or friend) work in the LAST 4 WEEKS. When answering, please ONLY consider the individual who provided the most care for this person when they were home due to their symptoms. This individual will be simply referred to as “caregiver” in the upcoming questions.

1. Is this caregiver currently employed or self-employed (i.e., working for pay)?

| A | Yes |
| --- | --- |
| B | No |

1. During the LAST 4 WEEKS, how many hours did the caregiver actually work?

____________Hours

1. During the LAST 4 WEEKS, how many hours did the caregiver miss from work **because of problems associated with this person’s symptoms of depression and/or anxiety**? *Include hours missed on days you did not work at all, days you went in late, days you left early, etc. because of your symptoms. Do not include time you missed to participate in this study.*

____________Hours

9. What is this caregiver’s typical personal monthly employment income from all sources, including self-employment or business ownership?

| 1 | Less than $1000 |
| --- | --- |
| 2 | $1000 - $1999 |
| 3 | $2000 - $2999 |
| 4 | $3000 - $3999 |
| 5 | $4000 - $4999 |
| 6 | $5000 - $5999 |
| 7 | $6000 - $6999 |
| 8 | $7000 -$9999 |
| 9 | $10,000 and over |
| 99 | Prefer not to answer |

*MAIN SURVEY – QUALITY OF LIFE ASSESSMENT (EQ5D-5L)*

Under each heading, please tick the ONE box that best describes this person’s health TODAY.

Mobility

|  | This person has no problems in walking about |
| --- | --- |
|  | This person has slight problems in walking about |
|  | This person has moderate problems in walking about |
|  | This person has severe problems in walking about |
|  | This person is unable to walk about |

Self-Care

|  | This person has no problems washing or dressing himself or herself |
| --- | --- |
|  | This person has slight problems washing or dressing himself or herself |
|  | This person has moderate problems washing or dressing himself or herself |
|  | This person has severe problems washing or dressing himself or herself |
|  | This person is unable to wash or dress himself or herself |

Usual Activities (e.g., work, study, housework, family or leisure activities)

|  | This person has no problems doing his or her usual activities |
| --- | --- |
|  | This person has slight problems doing his or her usual activities |
|  | This person has moderate problems doing his or her usual activities |
|  | This person has severe problems doing his or her usual activities |
|  | This person is unable to do his or her usual activities |

Pain/Discomfort

|  | This person has no pain or discomfort |
| --- | --- |
|  | This person has slight pain or discomfort |
|  | This person has moderate pain or discomfort |
|  | This person has severe pain or discomfort |
|  | This person has extreme pain or discomfort |

Anxiety/Depression

|  | This person is not anxious or depressed |
| --- | --- |
|  | This person is slightly anxious or depressed |
|  | This person is moderately anxious or depressed |
|  | This person is severely anxious or depressed |
|  | This person is extremely anxious or depressed |

-
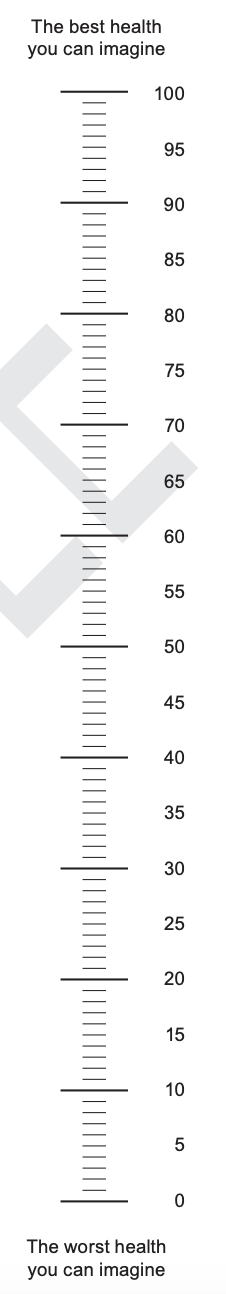
We would like to know how good or bad this person’s health is TODAY.
- This scale is numbered from 0 to 100.
- 100 means the best health you can imagine. 0 means the worst health you can imagine.
- Mark an X on the scale to indicate how this person’s health is TODAY.
- Now, please write the number you marked on the scale here: _______

**Appendix C. Data Quality Check and Cleaning**

Data was first thoroughly checked for straight line answers. No respondents provided straight line answers for questions pertaining to medication use or healthcare resource utilization (i.e., indicating that they had used all healthcare resources for the same number of times in the recall period). Data was next checked for skip logic. Eighteen respondents reported healthcare resource utilization (e.g., in-person visits, tele-visits, hospital resources, and diagnostic tests) despite responding to a prior question that they did not use any resources. All of these responses were recoded as missing for each respective sub-section. Four respondents reported working more than 80 hours in the preceding week. Given the unlikely nature of these weekly hours worked, these responses were also recoded as missing. One respondent stated that they’ve missed and worked more hours than there are in a particular week. This response was also recorded as missing.

**Supplementary Table 1. Demographic and Clinical Characteristics by Respondent Status.^1^**

|  | **Primary Respondents (N = 350)** | **Proxy Respondents (N = 79)** | **Significance** |
| --- | --- | --- | --- |
| **Mean Age** | 37.62 (SD: 11.60) | 51.09 (SD: 16.10) | **p < 0.001** |
| **Female (%)** | 187 (53.4%) | 37 (46.8%) | p = 0.289 |
| **Chinese (%)** | 286 (81.7%) | 66 (83.5%) | p = 0.702 |
| **Married (%)** | 176 (50.3%) | 48 (60.8%) | p = 0.092 |
| **Education Level (%)^3^** |  |  | **p < 0.001** |
| No Formal Education | 1 (0.3%) | 3 (3.8%) |  |
| Primary to Junior College | 159 (45.3%) | 45 (56.9%) |  |
| University and Above | 190 (54.4%) | 31 (39.2%) |  |
| **Employment Status (%)** |  |  | **p = 0.003** |
| Full-Time | 165 (47.1%) | 29 (36.7%) |  |
| Part-Time | 138 (39.4%) | 27 (34.2%) |  |
| Not Employed | 47 (13.4%) | 23 (29.1%) |  |
| **Monthly Income (%)** |  |  | **p < 0.001** |
| No Income | 47 (13.4%) | 32 (40.5%)^2^ |  |
| SGD 0 to SGD 1999 | 31 (8.9%) | 7 (8.9%)^2^ |  |
| SGD 2000 to SGD 4999 | 140 (40%) | 28 (35.4%)^2^ |  |
| SGD 5000 to SGD 9999 | 108 (30.9%) | 9 (11.4%)^2^ |  |
| SGD 10000+ | 24 (6.9%) | 3 (3.8%)^2^ |  |
| **Mean PHQ-4 Score** |  |  |  |
| Mean Total Score | 6.9 (SD: 3.3) | 5.2 (SD: 2.4) | **p < 0.001** |
| Mean Depression Sub-Score | 3.3 (SD: 1.7) | 2.4 (SD: 1.5) | **p < 0.001** |
| Mean Anxiety Sub-Score | 3.6 (SD: 1.6) | 2.8 (SD: 1.4) | **p < 0.001** |

1 Columns may not sum up to 100 due to rounding.

2 Monthly income for proxy adults was calculated using the average of the corresponding monthly income of the main respondents by occupation as indicated in the methods section.

3 This includes junior college, the Singapore-Cambridge General Certificate of Education Advanced Level (A-Level examination, polytechni education, diplomas, vocational training, and Institute of Technical Education (ITE) education. The A-Level is a national examination hel annually in Singapore. The examination is taken by school candidates on the completion of preuniversity education at junior colleges centralised institutions, and Integrate Programmes, and is also open to private candidates. ITE is a public vocational education institutio agency in Singapore that provides pre-employment training to secondary school graduates, and continuing education an training to working adults.

**Supplementary Table 2. Breakdown of average annual per capita costs by cost category and respondent status (in SGD).**

| **Cost Category** | **Primary Respondents (N = 350)** | **Proxy Respondents (N = 79)** |
| --- | --- | --- |
| ***Total Healthcare Cost*** | **1050 (3030)** | **1770 (3990)** |
| Medications | 40 (90) | 60 (100) |
| In-Person Visits | 380 (1240) | 720 (1640) |
| Televisits | 170 (770) | 350 (1120) |
| Diagnostic Tests | 290 (1110) | 470 (1850) |
| ED Visit and Hospitalization Costs | 170 (920) | 180 (520) |
| Percent with at least one ED Visit | 13% | 22% |
| Average Number of Admissions | 1.3 (0.5) | 1.1 (0.4) |
| Average Length of Stay per Admission | 3.3 (4.6) | 1.8 (1.3) |
| Percent with at least one Hospital Admission | 11% | 15% |
| ***Total Productivity Loss*** | **32580 (34970)** | **43700 (47540)** |
| Absenteeism | 4980 (9910) | 7870 (17160) |
| Days Missed from Work | 18 (38) | 24 (50) |
| Presenteeism | 28720 (29880) | 35830 (34700) |
| Presenteeism Score | 4.1 (2.7) | 4.0 (2.5) |
| Presenteeism Days from Work | 104 (91) | 114 (81) |

**Supplementary Table 3. Unsubsidized unit cost estimates of an demand for healthcare resources in Singapore.**

*Retrieved between June 2022 and August 2022. All costs include Goods and Services Tax (GST) of 7%.*

| Category | Unit Cost (SGD) | | Details | Sources |
| --- | --- | --- | --- | --- |
| Medications |  |  | |  |
| Daily Anti-Depressant or Daily Anti-Anxiety | $ 19.20 | Average monthly cost of the most common daily anti-depressant and/or anti-anxiety. | | Institute for Mental Health |
| As Needed Anti-Depressant or Anti-Anxiety | $ 24.00 | Average monthly cost of maintenance dose anti-depressant or anti-anxiety drug. Assume 50% utilization. | | Institute for Mental Health |
| As Needed for Insomnia | $ 24.00 | Average monthly cost of maintenance dose anti-depressant or anti-anxiety drug. Assume 25% utilization. | | Institute for Mental Health |
| Other Medication | $ 19.20 | Average monthly cost of the most common daily anti-depressant and/or anti-anxiety. | | Institute for Mental Health |
| Medication Not Listed Above | $ 19.20 | Average monthly cost of the most common daily anti-depressant and/or anti-anxiety. | | Institute for Mental Health |
| Physician Visits and Outpatient Procedures |  |  | |  |
| Polyclinic | $ 53.50 | Average of non-resident prices for General Practitioner consultations. | | Online payment schedules for SingHealth Polyclinics, National Healthcare Group, and National University Polyclinics |
| Private General Practitioner | $ 45.20 | Average of non-resident prices for Private General Practitioner consultations. | | Online payment schedules for Prohealth 24-hour, Central 24-hour Clinic (Woodlands), Thomson 24-Hr Family Clinic, and Intermedical 24-Hr Clinic |
| Psychiatrist - Public (Non-Subsidised) | $ 102.25 | Average of non-subsidized prices for initial and subsequent consultations for an adult at IMH. | | Institute for Mental Health |
| Psychologist - Private | $ 237.40 | Average of psychologist consultation fees (private practice, non-subsidized). | | Online payment schedules for Raffles Counselling Centre and Alliance Counseling Center |
| Social Worker | $ 95.00 | Average cost of a single one-hour counseling session with a social worker at IMH. | | Institute for Mental Health |
| Life Coach | $ 218.00 | Average of online session costs for career counseling. | | Online payment schedules for American Association of Singapore, Gary and Pearl International, Anagram Group, and Emunah Coaching |
| Diagnostic Tests |  |  | |  |
| EKG | $ 21.40 | Average cost of an EKG in Singapore. | | 1. Institute for Mental Health Singapore |
| EEG | $ 200.00 | Average cost of a neurology consult which would include an EEG. | | 1. Singapore General Hospital |
| CT/CAT Scan | $ 1,350.00 | Average price per CT/CAT scan at a public hospital. | | 1. Singapore General Hospital |
| MRI Imaging | $ 1,850.00 | Average price per MRI scan at a public hospital. | | 1. Singapore General Hospital |
| Emergency Department Visit | $ 127.00 | Average cost of an ED visit to IMH. | | 1. Institute for Mental Health Singapore |
| Hospitalization Costs Per Day | $ 555.00 | Average per daily ward and treatment fee for a one-bedded ward. | | 1. Institute for Mental Health Singapore |
